# Collagen Matrices Mediate Glioma Cell Migration Induced by an Electrical Signal

**DOI:** 10.3390/gels8090545

**Published:** 2022-08-29

**Authors:** Li Yao, Kimmy Tran, Diana Nguyen

**Affiliations:** Department of Biological Sciences, Wichita State University, 1845 Fairmount Street, Wichita, KS 67260, USA

**Keywords:** collagen, migration, electric field, glioma, astrocyte

## Abstract

Glioma cells produce an increased amount of collagen compared with normal astrocytes. The increasing amount of collagen in the extracellular matrix (ECM) modulates the matrix structure and the mechanical properties of the microenvironment, thereby regulating tumor cell invasion. Although the regulation of tumor cell invasion mainly relies on cell–ECM interaction, the electrotaxis of tumor cells has attracted great research interest. The growth of glioma cells in a three-dimensional (3D) collagen hydrogel creates a relevant tumor physiological condition for the study of tumor cell invasion. In this study, we tested the migration of human glioma cells, fetal astrocytes, and adult astrocytes in a 3D collagen matrix with different collagen concentrations. We report that all three types of cells demonstrated higher motility in a low concentration of collagen hydrogel (3 mg/mL and 5 mg/mL) than in a high concentration of collagen hydrogel (10 mg/mL). We further show that human glioma cells grown in collagen hydrogels responded to direct current electric field (dcEF) stimulation and migrated to the anodal pole. The tumor cells altered their morphology in the gels to adapt to the anodal migration. The directedness of anodal migration shows a field strength-dependent response. EF stimulation increased the migration speed of tumor cells. This study implicates the potential role of an dcEF in glioma invasion and as a target of treatment.

## 1. Introduction

Glioblastoma is an aggressive type of brain cancer, and its malignancy is the result of the invasive behavior of tumor cells [1,2]. The extracellular matrix molecules regulate glioma cell migration and invasion. In tumor tissue, glioma cells produce type I and type IV collagens that increase the collagen content [3]. Collagen in the extracellular matrix (ECM) provides scaffolds for glioma cell migration [4,5]. Collagen also alters the tissue’s mechanical strength and therefore enhances tumor cell invasion [3]. In one in vivo study, collagen fibers were detected by Masson’s trichrome, picrosirius red, and second harmonic generation microscopy in mouse brain glioma. The study showed the variation in collagen fiber density at different regions of the glioma [6]. To understand the effect of collagen fibers on tumor cell invasion, various types of tumor cells have been tested for their migration in different concentrations of collagen gels. The concentration range of the collagen solutions in these studies was between 1 mg/mL and 6 mg/mL [7,8,9,10]. One in vitro study has attempted to characterize glioma cell migration behavior in a collagen gel. When the tumor aggregates were grown in collagen hydrogels ranging from 0.5 mg/mL to 2.0 mg/mL, the cell migration velocity increased in gels with a high collagen concentration compared with those having a low collagen concentration [11]. Although the correlation of the collagen fiber density in a glioma and collagen hydrogel in an in vitro study has not been established, testing the glioma cell migration at a higher collagen concentration that reflects the possible high collagen fiber density in vivo will provide more information on glioma cell migration.

In our previous study, the collagen gel viscosity was chemically increased by crosslinking the collagen with eight-arm PEG succinimidyl glutarate (hexaglycerol) (8S-StarPEG), showing that the glioma cell migration speed decreased when cells were grown in the crosslinked collagen gel compared with non-crosslinked collagen gel [12]. Collagen can generate a high-viscosity solution when its concentration is increased to 10 mg/mL. However, information relative to tumor cell invasion in collagen gels with a higher concentration between 3 mg/mL and 10 mg/mL is lacking. Testing the glioma cell migration in these gels will help to explore the invasion behavior of tumor cells.

Although ECM molecules are major factors that modulate tumor cell migration and have been extensively investigated, the electrotaxis of tumor cells has attracted increasing research interest [13,14,15]. Electrical activity has been detected in developing nervous tissue. The endogenous electric field (EF) was measured at the neural plate and neural fold stages of axolotl embryos using a vibrating probe, and this may contribute to the development of the central nervous system (CNS) [16,17,18]. In further research, a wound-generated EF was detected in the rat cornea, guiding the wound-healing process and peripheral nerve growth [19]. The rapid growth of glioma cells causes tissue necrosis [20] and damage to the local tissue structure [21], and may induce an EF at the wounded epithelial tissue, though this has not been proven yet. The electrotaxis of tumor cells has attracted great research interest because the endogenous EF may play a role in regulating tumor cell invasion, and an applied EF may be used to control this. In one study, human glioma cell lines were cultured as differentiated and glioma stem cells (GSCs) and these cells were subjected to EF stimulation. The cells showed direction migration in an EF [14]. Although the EF-guided glioma cell migration on a two-dimensional (2D) dish surface was reported [14], the growth of glioma cells in a three-dimensional (3D) collagen hydrogel creates a relevant tumor physiological condition environment for the study of tumor cell invasion.

Studies have shown the impact of tumor treating fields (TTFields), which are alternating electric fields (AEFs) and are approved by the FDA for glioma treatment [22,23]. Noninvasive technology for anticancer therapy is an emerging approach to treat glioma. However, no clinical trial has been performed to study the effect of transcranial direct current stimulation (tDCS) on glioma therapy. It was reported that tDCS treatment may lead to a reduction in glioma perfusion and metabolism [24]. The study of direct current electric field (dcEF) for guided glioma cell migration based on 3D culturing will contribute to the knowledge of the impact of direct current stimulation on the invasive behavior of glioma cells.

In the present study, human glioma cells were grown in different concentrations of collagen gel (3 mg/mL, 5 mg/mL, and 10 mg/mL). Cell migration was observed and recorded with a time-lapse microscope. As a control, astrocyte migration in the collagen matrix was also investigated. Dissociated glioma cells were grown in collagen gel, and a dcEF was applied to the cells in the gels. The cell migration pattern was then characterized by analyzing the time-lapse imagery.

## 2. Results

### 2.1. Glioma Cells, Fetal Astrocytes, and Adult Astrocytes Showed High Migration Velocity in Low-Concentration Collagen Hydrogels

**Figure 1:**
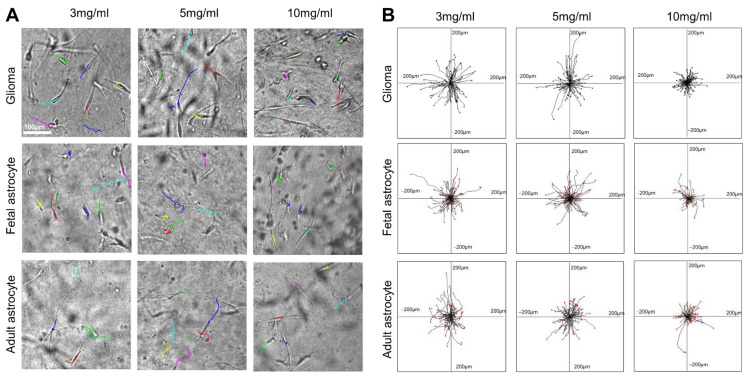
Cell migration in collagen hydrogels of different concentrations. (**A**) Human glioma cells (Supplemental Videos S1–S3), fetal astrocytes (Supplemental Videos S4–S6), and adult astrocytes (Supplemental Videos S7–S9) showing elongated shapes in collagen gels. Migration was tracked based on time-lapse recording and labeled with lines. (**B**) Combined cell migration tracks of glioma cells, adult astrocytes, and fetal astrocytes. The center of each frame represents the origin position of each cell migration. Each line represents the migration projection of each cell. The lines of each frame represent combined cell migrations from at least three gels, as each study was repeated at least three times.

**Figure 2:**
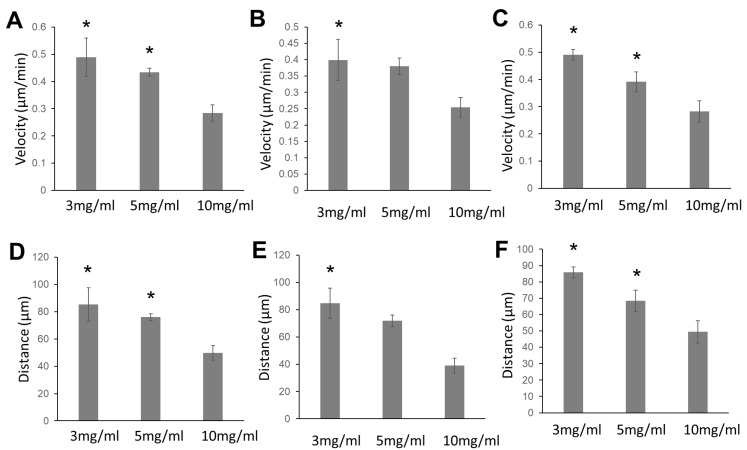
Quantification of cell migration showing the reduction in cell motility of glioma cells (**A**,**D**), adult astrocytes (**B**,**E**) and fetal astrocytes (**C**,**F**) in high-collagen hydrogels (10 mg/mL) compared with low-concentration collagen gels (3 mg/mL and 5 mg/mL). This comparison was performed using one-way ANOVA *, *p* < 0.05, vs. collagen gel (10 mg/mL).

In this study, we observed the migration of glioma cells, adult astrocytes, and fetal astrocytes in collagen hydrogels of three different concentrations (3 mg/mL, 5 mg/mL, and 10 mg/mL) (Figure 1) (Supplemental Videos S1–S9). Most glioma cells and astrocytes in the collagen gels showed an elongated bipolar or unipolar morphology. The long leading processes and the process tips are dynamic structures that direct the migration of the whole cell (Figure 1A). The cell migration velocity was calculated from the full cell migration distance during the three-hour recording time. All cell types showed the highest migration velocity in 3 mg/mL collagen hydrogels (Figure 2). For all cell types, the migration speed in 5 mg/mL collagen hydrogels decreased slightly when compared with that in 3 mg/mL collagen hydrogels. However, the reduction in the velocity was not significant. For all cell types, the cell migration velocity was reduced significantly in 10 mg/mL collagen gels compared with that in 3 mg/mL collagen hydrogels.

### 2.2. The Electric Field Directed Glioma Cell Migration in the Collagen Gels

**Figure 3:**
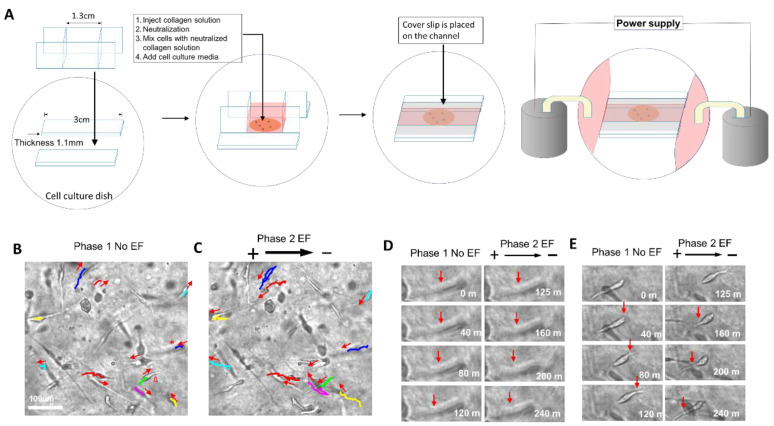
Anodal migration of glioma cells in an applied electric field. (**A**) Setup of cell culture chamber for cell culturing in collagen gels subjected to EF stimulation. (**B**) Cell random migration for two hours (Supplemental Video S10). (**C**) Migration of the same group of cells in (B) recorded for 2 additional hours after the electric field. The migration of cells is labeled with different lines. Each color indicates the same cell in random cell migration (B) and migration in the EF (C). The colored lines show the cell migration pathways. The lines show the migration trajectory of phase 1 in (B), while they show the full trajectory of phase 1 and phase 2 in (C). The red arrows show the cell migration directions at the end of phase 1 and phase 2. (**D**) Random migration showing one cell (labeled with star in (B) migrating toward the left for two hours. After EF was switched on, the cell continued toward the left, in the direction of the anode. (**E**) Random migration showing one cell (labeled with triangle in (B) migrating toward the right for two hours. After EF was switched on, the cell swapped the leading and trailing processes, and migrated toward the left, in the direction of the anode. The red arrows in (D,E) show the extension of the leading processes of the cells.

This study showed how the EF guided the migration of human glioma cells in collagen hydrogels. In the experimental design, random cell migration was recorded for two hours, and then an EF power supply was switched on and the cell migration was recorded for another two hours. Therefore, the migration of the same group of cells was compared directly under conditions without EF stimulation and with EF stimulation. After the cells were subjected to the stimulation of 200 mV/mm, the random migration pattern of most cells changed to clear anodal migration (Figure 3B,C).

The migration of two typical cells that are labeled with a star and triangle in Figure 3B is highlighted in Figure 3D,E respectively. One cell migration direction was toward the left in the phase of random migration (phase 1, without EF stimulation) (Figure 3D). The leading process extended to the left and led the migration. After the EF power supply was turned on, the leading process continued to extend toward the left and led the movement to the left, which is in the direction of the anodal pole. For the second cell, the cell migrated to the right in the phase of random migration, which was guided by the leading process extending to the right (Figure 3E). After the EF power supply was switched on, the leading and trailing processes swapped directions. The leading process on the right shrank and the trailing process extended toward the left and led the cell movement to the left, which is in the direction of the anodal pole.

### 2.3. The Increase in Field Strength Enhanced Anodal Migration Directedness

**Figure 4:**
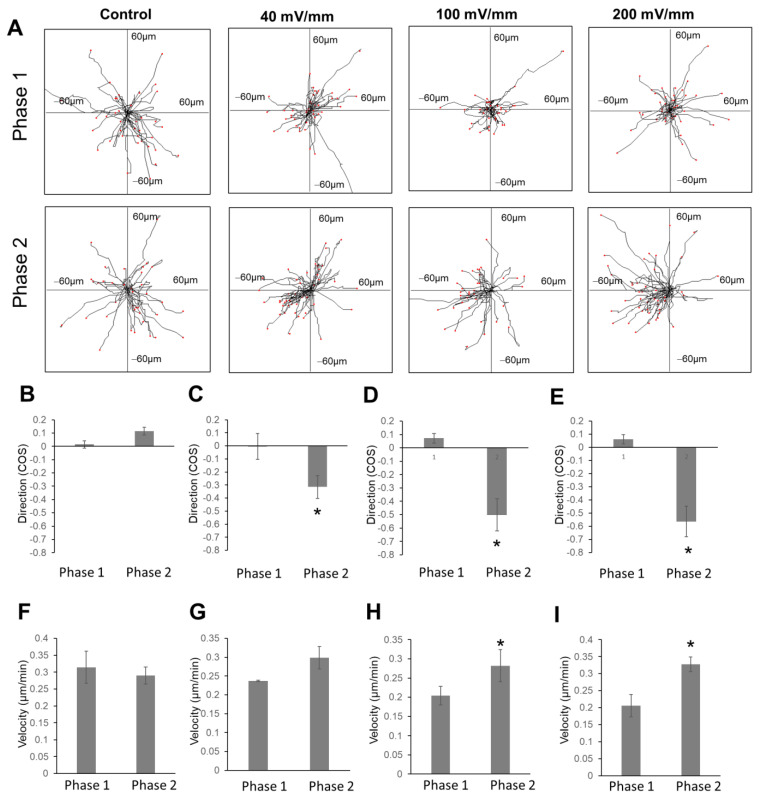
Quantification of cell migration directedness and velocity in EF. (**A**) Cell migration tracks showing the directional migration of glioma cells in an EF (100 mV/mm and 200 mV/mm). (**B**–**E**) Migration directedness and (**F**–**I**) speed of the same group of cells before and after EF was switched on, directly compared using paired *t*-test *, *p* < 0.05, vs. random cell migration for two hours.

Quantification of cell migration directedness and velocity was performed (Figure 4). For control groups, the directedness of glioma cell migration in the first phase (first two hours) and second phase (second two h) was 0.01 ± 0.02 and 0.11 ± 0.02, respectively (Figure 4B). For the 40 mV/mm group, the directedness of glioma cell migration in the first phase (with EF) and second phase (without EF) was 0.00 ± 0.09 and −0.31 ± 0.08, respectively. For the 100 mV/mm group, the directedness of glioma cell migration in the first and second phases was 0.07 ± 0.03 and −0.50 ± 0.011, respectively. For the 200 mV/mm group, the directedness of glioma cell migration in the first and second phases was 0.06 ± 0.03 and −0.56 ± 0.11, respectively. For the 40 mV/mm, 100 mV/mm, and 200 mV/mm groups, the difference in directedness for the first and second phases was significant (*p* < 0.01).

### 2.4. EF Stimulation Increased Glioma Cell Migration Velocity in the Collagen Hydrogels

The cell migration velocity in this study was calculated from the full cell migration distance during the two-hour recording time of each phase. The migration velocity in the 100 mV/mm and 200 mV/mm groups showed an increase when comparing the first and second phases, and the difference was statistically significant (*p* < 0.01). For the 100 mV/mm group, the migration speeds in the first and second phases were 0.20 ± 0.02 µm/h and 0.28 ± 0.04 µm/h, respectively. For the 200 mV/mm group, the migration speeds in the first and second phases were 0.20 ± 0.03 µm/h, and 0.32 ± 0.02 µm/h, respectively.

### 2.5. Reversal of EF Polarity Reversed the Cell Migration Direction

**Figure 5:**
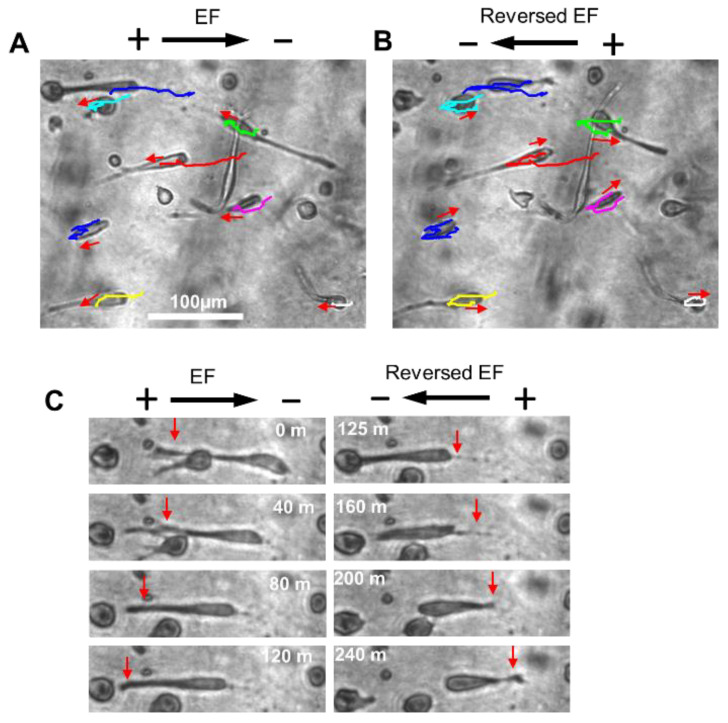
EF poles reversing cell migration direction (Supplemental Video S11). (**A**) Cells migrating toward the left, in the direction of the anode. (**B**) After EF polarity was switched to the right, the cells switched and migrated to the new anode (right). (**C**) The leading and trailing processes switched after the EF poles switched.

**Figure 6:**
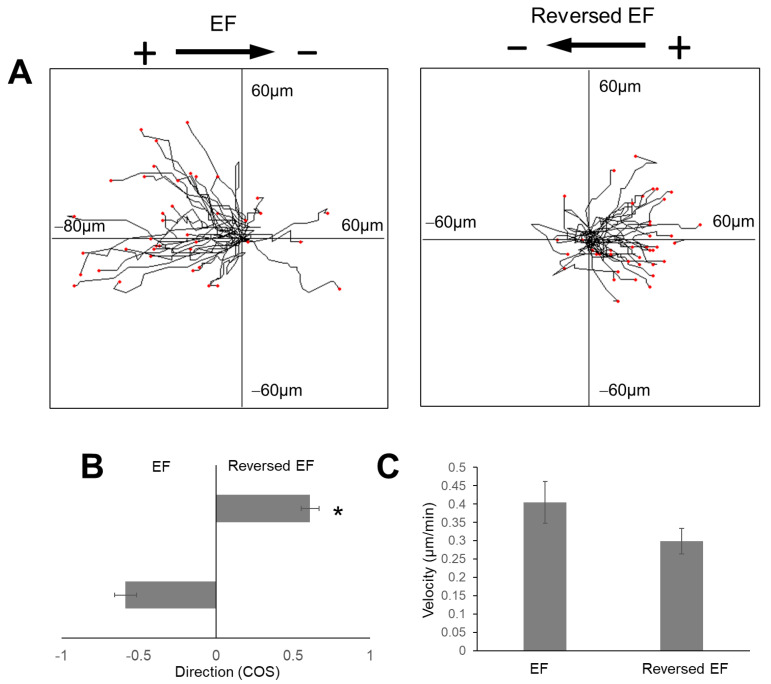
Quantification of cell migration after switching EF polarity. (**A**) Projections of cells before and after EF polarity switch. (**B**) EF polarity change switching cell migration directedness. (**C**) Cell migration speed difference was not statistically significant. *, *p* < 0.05, vs. cell migration direction of phase 1.

To confirm the anodal migration of glioma cells in collagen hydrogels, the cell migration polarity was reversed in the second phase. Figure 5A shows the reversal of the cell migration direction of the group of cells. One typical cell migration in the EF is shown in Figure 5B, where the cell moved to the anode on the left, and migration was led by the long leading process. After the EF power supply was switched on, the leading process shrank, and the trailing process grew longer and led the cell movement toward the new anode on the right. The directedness of cell migration in the first and second phase was 0.58 ± 0.07 and 0.60 ± 0.05, respectively (Figure 6). This observation suggested that the cells moved to the new anodal pole after the polarity was switched.

## 3. Discussion

Studies have shown the impact of the ECM mechanical properties on tumor cell invasion. In one previous study, a few human glioma cell lines (U373-MG, U87-MG, U251-MG, SNB19) and one rat glioma cell line (C6) were grown on fibronectin-coated polymeric substrates, which had different mechanical rigidities. The tumor cells spread extensively and showed higher migration speed on the substrate with higher rigidity compared to the substrate with lower rigidity [25]. However, a three-dimensional cell culture can create an environment that mimics better the native tumor growth condition. One study tested U87 glioma cell growth in collagen gels (0.5 mg/mL, 1.0 mg/mL, 1.5 mg/mL, and 2.0 mg/mL). The cell migration distance in the high-concentration collagen gel was longer than that in the low-concentration collagen gel, within the concentration range of 0.5 mg/mL and 2 mg/mL. The mentioned study suggested that the invasion in the high-concentration collagen gel was more effective [11]. H1299 cells (lung cancer cell line) were grown in collagen gels with concentrations of 2.5, 4, and 6 mg/mL. The higher collagen concentration of the hydrogel was correlated with higher gel stiffness [10]. The cells showed lower motility in collagen gels with a higher concentration compared with that in collagen gels with a lower concentration [10]. In another previous study, we compared glioma cell migration on a cell culture plate to that in collagen gels (5 mg/mL). Cell migration in the collagen gels showed higher velocity than that on the cell culture plate. In the previous study, the gel viscosity was increased by crosslinking the gel with 8S-StarPEG. It was shown that there was a lower cell migration speed in the crosslinked gel (5 mg/mL) compared with that in the non-crosslinked gel (5 mg/mL) [12]. The present investigation studied the human glioma cell and astrocyte migration in collagen hydrogels with different concentrations (3 mg/mL, 5 mg/mL, and 10 mg/mL). The study aimed to explore how collagen concentration affects the motility of glioma cells and astrocytes. We observed that the tumor cells, adult astrocytes, and fetal astrocytes showed lower cell migration velocity in the high-concentration collagen hydrogel (10 mg/mL) compared to the low-concentration gels (3 mg/mL and 5 mg/mL). Our previous study showed that the migration velocity of U87 cells and A172 cells in crosslinked collagen hydrogels was lower than that in non-crosslinked gels. The present study showed that the high collagen density reduced the tumor cell migration velocity, in turn supporting our previous observation. Our study also observed similar results for H1299 cell migration in collagen gels as described above [10]. These studies indicate that there may be a favorable collagen density for tumor cell migration and invasion.

Electric fields have demonstrated a significant effect on guided neural cell migration. We reported that the migration of the hippocampal neurons [26], Schwann cells [27], oligodendrocyte progenitor cells (OPCs) [28], and neural stem cells [28,29] can be guided by an applied EF. The neurons and neural stem cells were reported to migrate to the cathodal pole, while the glial cells, including Schwann cells, OPCs, and astrocytes, migrated to the anodal pole. Although these studies observed the different responses of the glial cells and neuronal cells to the applied EF stimulation, the mechanism that controls the migration direction of the cells is not clear. A previous study showed that the glioma cell migration can be guided to move toward the anodal pole on a 2D cell culture dish [14,30]. Interestingly, glioma originates from glial cells, and astrocytes and glioma cells all showed anodal migration. Further study is needed to explore the mechanism of the EF-guided migration direction of these cells. Cell migration in a 3D cell condition has been attempted in previous studies. Dictyostelium cells that were grown in agarose gel were subjected to EF stimulation and showed directional migration toward the cathode [31]. Human embryonic stem cell (H7 cell line) aggregates were grown in Matrigel and subjected to EF stimulation. The cells that migrated from aggregates responded to EF stimulation and moved toward the anode [32]. In another study, glioma cell lines (U251 and U87) formed spheroidal aggregates, and the aggregates were grown in Matrigel. The cells were subjected to EF and showed anodal migration [13]. In the present study, glioma cells that were isolated from a patient were investigated for their migration in collagen hydrogels. The tracking of dissociated cell migration provided a platform to visually characterize individual cell migration and the response to EF stimulation. We found that glioma cell migration can be guided toward the anodal pole by EF in a 3D environment. The anodal migration of glioma cells on culture dishes was reported previously [14]. Similar to the migration of glioma cells [14] and other neural cells [26,28,29] on culture dishes, the cell migration directedness increased when the strength of the EF voltage increased. Interestingly, we also found that the cell migration velocity can be increased by EF stimulation after the cells are subjected to field strengths of 100 mV/mm and 200 mV/mm. As an approach involving alternating electrical fields, tumor-treating fields (TTFields) have been shown to improve the survival rate of patients with glioblastoma [33]. In vitro studies showed that the alternating electrical fields inhibited glioma cell proliferation [33,34]. Our present study demonstrated the guidance effect of a direct current electric field on glioma cell migration on a 3D model. The effect should be confirmed by further in vivo study.

In this study, we observed that the typical cell morphology changed during migration in collagen gels. The glioma cells and astrocytes showed an elongated morphology. The leading processes of the cells guided the direction of the cell migration in collagen gels by altering the direction or generating branch. In an electric field, tumor cells maintained a similar morphology to that in collagen gels without EF stimulation. Typically, cells can change their migration direction by swapping the leading and trailing processes when the EF polarity is switched. This study suggests that the EF can orient tumor cell migration by affecting the cell processes.

## 4. Conclusions

Human glioma cells, fetal astrocytes, and adult astrocytes showed higher motility in a low concentration of collagen hydrogel (3 mg/mL and 5 mg/mL) than in a high concentration of collagen hydrogel (10 mg/mL). Human glioma cells grown in collagen hydrogels responded to electric field (EF) stimulation and migrated to the anodal pole. The tumor cells altered their morphology in the gels to adapt to the anodal migration. The directedness of anodal migration shows a field strength-dependent response. EF stimulation increased the migration speed of tumor cells. Reversal of the EF polarity can reverse the glioma migration direction in collagen hydrogels. The study showed that collagen can serve as a matrix for electric signal-guided tumor cell migration. The study indicates the potential of electric fields in glioma invasion and as a target of tumor treatment.

## 5. Materials and Methods

### 5.1. Cell Culture in Collagen Hydrogels

Primary human glioma cells (HCM-BROD-0002-C71, ATCC, Manassas, VA, USA) were cultured using conditioned medium (256–100 Propagenix Inc, Gaithersburg, MD, USA). Human adult astrocytes (Cell Applications, Inc., San Diego, CA, USA) were cultured with human astrocyte growth medium (Cell Applications, Inc., San Diego, CA, USA). Human fetal astrocytes (ScienCell Research Laboratories, Carlsbad, CA, USA) were cultured using astrocyte medium (ScienCell Research Laboratories, Carlsbad, CA, USA).

Type I collagen, extracted from bovine Achilles tendon (Stroots Locker, Goddard, KS, USA), was dissolved in acetic acid to prepare collagen solutions of 3 mg/mL, 5 mg/mL, and 10 mg/mL. To generate the 3D cell cultures, glioma cells, human adult astrocytes, and human fetal astrocytes were seeded inside the collagen hydrogels. Collagen solution (400 µL) in the wells of a 48-well plate was neutralized with NaOH solution (1 M) and phosphate-buffered saline (PBS) solution (10× [12]. Then, 40,000 cells of each cell type were mixed with the neutralized collagen solution. Additional pre-warmed medium was then gently added into the wells with the collagen gel and cell mixture. The cell culture plate was then transferred to an incubator at 37 °C with 5% CO_2_.

### 5.2. EF Application for Glioma Cells Cultured in Collagen Hydrogels

A special chamber was made to study the migration of glioma cells subjected to EF stimulation in collagen hydrogels. In a culture dish, a plastic channel was constructed with the dimensions of 30 mm (length) × 0.8 mm (width) × 1.1 mm (depth). A glass frame with chamber dimensions of 13 mm (length) × 12 mm (height) × 0.8 mm (width) was placed in the chamber (Figure 3A). A collagen solution (200 µL, 5 mg/mL) was placed in the chamber and neutralized, as described above. Then, 40,000 glioma cells were mixed gently with the collagen gel in the chamber. After culturing for 24–48 h, the glass frame was removed, and direct current electric fields (dcEFs) were applied to the cultured cells. The cell culture medium was connected to Steinberg’s solution in beakers using agar salt [26]. Electric power was applied to the cells through silver chloride electrodes placed in the beakers. Then, direct current fields (40 mV/mm, 100 mV/mm, and 200 mV/mm) were applied to the cells. In the control study, no EFs were applied to the cells.

### 5.3. Time-Lapse Imaging for Recording Cell Migration

The cell culture plate and electric field chamber were placed on the stage of a microscope (Zeiss Axio Observer microscope, White Plains, NY, USA), which was included in a plastic box for cell migration recording. The temperature of the plastic box was set at 37 °C and supplied with CO_2_ (5%). Time-lapse image recording was performed using the ZEN 2011 program installed in the microscope to control the capturing of time-lapse imaging. A digital camera (AxioCam MRm Rev.3 with FireWire, White Plains, NY, USA) was used to take images. The migration of the cells was recorded for three hours for cell migration in collagen gels of different concentrations. For cells subjected to EF stimulation, cell migration was recorded for two hours for the control condition without EF stimulation. The cell migration was then continuously recorded for an additional two hours after the EF was activated. A separate group of experiments were performed to confirm the guidance effect of EF on cell migration direction. In this research group, cell migration subjected to EF stimulation was recorded for two hours, and then the migration was continuously recorded for two hours after the EF polarity was reversed. During the recording period, the images were captured every five minutes. Each gel made in one 48-well plate well or in one EF chamber was used to capture only one time-lapse image of cell migration. At least three recordings were performed for each experimental group.

### 5.4. Cell Migration Analysis

The cell migration in time-lapse images was manually tracked using NIH ImageJ software (National Institutes of Health, Bethesda, MD, USA). The cell tracking data were further quantified using the Chemotaxis and Migration Tool 2.0 (ibidi USA, Fitchburg, WI, USA). Cell migration velocity and distance were analyzed in collagen gels with different concentrations. For cell migration under EFs, the method was previously reported [26]. The cell migration direction was expressed as the cosine of the angle. The directedness of each experimental group was calculated from the equation (∑icosθ)/n, where n is the cell number of all replicates. The cell migration velocity was calculated from the accumulated cell migration distance during the recording time.

### 5.5. Statistical Analysis

The quantified data are all expressed as mean ± standard deviation. Statistical analysis was performed using a two-tailed Student’s *t*-test by Excel and ANOVA by SPSS, with a *p*-value of 0.05 considered to be statistically significant.

## Supplementary Materials

The following are available online at https://www.mdpi.com/article/10.3390/gels8090545/s1. Supplemental Video S1: glioma cell migration in 3 mg/mL collagen gel. Supplemental Video S2: glioma cell migration in 5 mg/mL collagen gel. Supplemental Video S3: glioma cell migration in 10 mg/mL collagen gel. Supplemental Video S4: fetal astrocyte migration in 3 mg/mL collagen gel. Supplemental Video S5: fetal astrocyte migration in 5 mg/mL collagen gel. Supplemental Video S6: fetal astrocyte migration in 10 mg/mL collagen gel. Supplemental Video S7: adult astrocyte migration in 3 mg/mL collagen gel. Supplemental Video S8: adult astrocyte migration in 5 mg/mL collagen gel. Supplemental Video S9: adult astrocyte migration in 10 mg/mL collagen gel. Supplemental Video S10: EF-directed glioma cell migration in collagen gel. Supplemental Video S11: EF polarity switch directing glioma cell migration in collagen gel.
